# *QuickStats*: Percentage*^,†^ of Men and Women Aged 25–49 Years Who Spent at Least One Night in the Past 12 Months at an Alternate Location Because They Did Not Have a Permanent Place To Stay, by Type of Location^§^ — National Survey of Family Growth, United States, 2017–2019

**DOI:** 10.15585/mmwr.mm7028a4

**Published:** 2021-07-16

**Figure:**
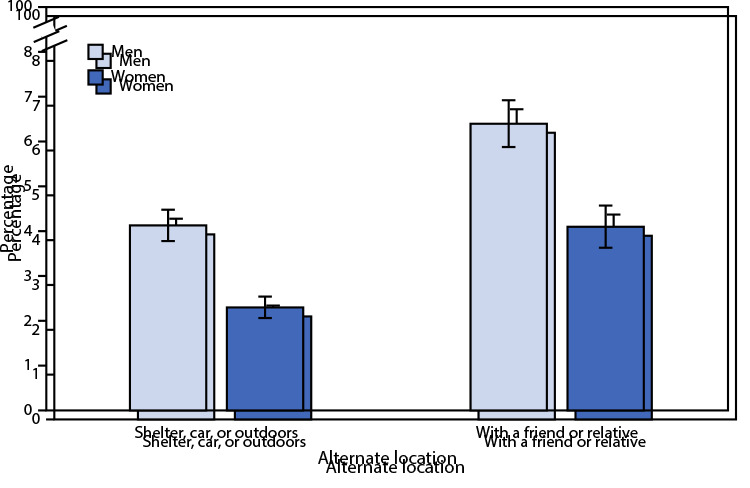
Among adults aged 25–49 years, a higher percentage of men (4.1%) than women (2.3%) stayed at least overnight in a shelter or car or outdoors in the past 12 months because they did not have a permanent place to stay. A higher percentage of men (6.4%) than women (4.1%) stayed at least overnight with a friend or relative in the past year. Among both men and women, the percentage who stayed at least overnight with a friend or relative was higher than the percentage who stayed at least overnight in a shelter or car or outdoors.

**Source:** National Survey of Family Growth, 2017–2019. https://www.cdc.gov/nchs/nsfg/index.htm

